# Reducing Upper-Limb Muscle Effort with Model-Based Gravity Compensation During Robot-Assisted Movement

**DOI:** 10.3390/s25103032

**Published:** 2025-05-12

**Authors:** Leigang Zhang, Hongliu Yu, Desheng Li

**Affiliations:** 1 Institute of Rehabilitation Engineering and Technology, Shanghai University of Science and Technology (USST), Shanghai 200093, China; lgzhang@usst.edu.cn (L.Z.); yhl98@hotmail.com (H.Y.); 2 Shanghai Huizhikang Intelligent Technology Co., Ltd., Shanghai 201800, China

**Keywords:** arm weight compensation, upper limb rehabilitation robot, muscle activation

## Abstract

Clinical research has demonstrated that stroke patients benefit from active participation during robot-assisted training. However, the weight of the arm impedes the execution of tasks and movements due to the functional disability. The purpose of this paper is to develop a gravity compensation strategy for an end-effector upper limb rehabilitation robot based on an arm dynamics model to reduce the arm’s muscle activation level. This control strategy enables real-time evaluation of arm gravity torques based on feedback from upper limb kinematic parameters. The performance of the proposed strategy in movement tracking is then compared to that of other types of weight compensation strategies. Experimental results demonstrate that compared to movements without compensation, the mean activation levels of arm muscles with the proposed strategy showed a significant reduction (*p* < 0.05), except for activation of the triceps. Furthermore, the proposed strategy provides superior performance in reducing the arm muscle’s effort compared to the position-varying weight compensation strategy. Therefore, with the proposed strategy, the end-effector rehabilitation robot might improve participation in robot-assisted rehabilitation training, as well as the usability and feasibility of the rehabilitation or assistive robot.

## 1. Introduction

Clinical research has demonstrated that rehabilitation robots have the advantage of conducting repetitive and task-oriented movements, which have a positive effect on patients with motor disabilities [1,2]. However, achieving movement and task execution is significantly affected by the arm’s weight in stroke patients because of their weak muscles [3]. Traditional position controllers ignore the voluntary effort and focus on trajectory-tracking accuracy for patients with partial motor control. Nevertheless, subjects’ active participation in rehabilitation training can significantly facilitate therapeutic efficacy [4]. Complete or partial arm weight compensation enables patients to engage more actively in rehabilitation training and perform tasks that are highly relevant to activities of daily living [5]. Arm weight compensation has been proven to be an important factor in enabling patients to perform tasks that require longer reaching distances and an increased workspace [6]. Furthermore, robot-assisted rehabilitation training with gravity compensation has demonstrated benefits in reducing interactions or task execution caused by gravity [7] and can increase active participation without changing muscle activation patterns.

Several rehabilitation robots or devices with partial or complete arm weight compensation have been developed, which can be divided into three categories. The first is weight compensation using mechanisms [5,8,9]. These devices provide arm weight support through fixed or artificially adjusted compensation, such as an elastic band mechanism [5], a multilink mechanical structure [8], or a balanced spring mechanism [9]. These devices can compensate for weight at different levels but are unable to apply other strategies. The second is weight compensation by a passive system. Arm weight is compensated for by adding a separate device to the rehabilitation robot system. For example, the MIT-Manus applies a wrist support module to achieve gravity compensation of the patient’s arm during planar motion [10]. A rope suspension system is adopted in the Gentle/S to achieve arm weight compensation [11]. Using a rope that is attached to a spring, Freebal achieves gravity compensation at various levels while satisfying the subject’s motion tracking in both the horizontal and vertical planes [12]. The third category is arm weight compensation through end-effector-based and exoskeleton-based robots. Robot controllers are usually adopted in these kinds of systems to actively compensate, either totally or partially, for the arm’s weight. For instance, the exoskeleton system Harmony enables upper limb gravity compensation by accounting for the arm’s weight in the robot’s control scheme [13]. Just et al. developed a predictive model of upper limb gravity torque based on the position and mass of the arm’s center of mass and then tested this on ARMin [14,15]. Crocher et al. reduced the upper limb to a two-link model, used inverse kinematics to derive the arm’s motion characteristics, and initially verified their gravity compensation method on EMU [16]. Furthermore, other rehabilitation robotic systems, including a soft rehabilitation robot [17], a rope-driven upper limb rehabilitation robot [18], and a wearable robot [19], have shown promising results in arm weight compensation.

Since the gravity torque of the arm is highly dependent on the kinematic position of the upper arm and forearm during movement, fixed weight compensation (mechanism) or varying elastic compensation strategies, such as Armeo Spring and Saebo, cannot consider the position-coupling effect. Passive robotic weight compensation systems, such as Gentle/S and Freebal, are unable to provide a quantitative estimation of arm weight, although they can implement arm gravity compensation at various levels. In active robotic weight compensation systems, either an equivalent gravity approach (based on the upper limb’s physical characteristics) or an adaptive approach is commonly employed to accomplish weight compensation. Song Rong et al., for example, used the moment balance method to evaluate the equivalent gravity of the arm based on the center location of each upper limb joint during movement. The Zurich team calculates the equivalent weight to be compensated for based on the weight of the various arm segments. Although equivalence-based arm weight estimation or adaptive compensation methods [17,20] can achieve positive weight compensation results, it is difficult for these methods to provide theoretical guarantees of estimated disturbance accuracy. Evaluating the gravity torques based on the arm dynamics model can provide a theoretical guarantee, although only a few related studies have been reported. Crocher et al. conducted a preliminary gravity compensation analysis based on an arm model; however, only preliminary simulation validation experiments using a manipulator were performed. In addition, further performance validation of the weight compensation strategy, such as at the level of muscle activation, has yet to be performed.

Therefore, the purpose of this paper is to develop a gravity compensation strategy based on varying the arm position for an end-effector rehabilitation robot. A model-based gravity torque estimation model is proposed to evaluate the real-time upper limb gravity torque. Then, the performance of the proposed strategy is compared with two other weight compensation strategies (e.g., without compensation and position-varying weight compensation) during human–robot cooperation movement tracking in different directions. The activation of the subjects’ six muscles in different directions was observed and used to assess different weight compensation strategies with an end-effector upper limb rehabilitation robot. In comparison to existing works, the distinguishing novelties and contributions of this work are:

(1) Compared with other upper limb weight compensation methods, such as the position-varying compensation strategy, the weight compensation strategy based on the arm dynamics model is adopted in this paper;
(2) In the weight compensation method proposed in this paper, the weight estimation of the arm is calculated based on the real-time joint data of the arm, rather than using the equivalent weight estimation method;
(3) In this paper, an arm weight compensation experiment based on a point-to-point task was carried out to observe the activation degree of the upper limb muscle group, and the performance of the proposed weight compensation method was compared with that of the method without compensation and the position-varying compensation method.

The remainder of this paper is organized as follows: Section 2 describes the weight compensation strategy based on the arm dynamics model. Validation experiments and the results of the proposed strategy are detailed in Section 3 and Section 4, respectively. Finally, Section 5 and Section 6 provide the discussion and conclusion.

## 2. Arm Gravity Compensation Method

### 2.1. Arm Model Reconstruction

**Figure 1:**
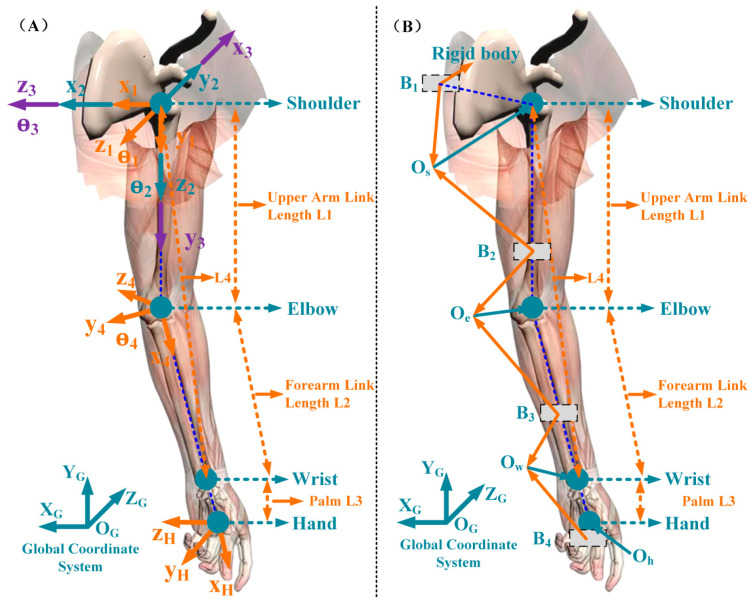
Kinematic model of the upper limb. (**A**) 4-DOF kinematic model; (**B**) schematic diagram of arm motion reconstruction.

As illustrated in Figure 1A, a simplified four degrees-of-freedom (DOF) upper limb model was adopted. The model’s DOF was assigned as follows: three DOF ($\theta_{1}∼\theta_{3}$) for the shoulder joint, and one DOF ($\theta_{4}$) for the elbow joint. The joints in the model were mainly established in accordance with the ISB [21], where $L_{4}$ is the distance between the wrist and shoulder joint centers, $L_{1}$ and $L_{2}$ are the lengths of the upper arms and forearms, and $L_{3}$ is the length between the hand and wrist.

The arm’s kinematic parameters were solved using a motion capture system to reconstruct the upper limb model. As seen in Figure 1B, a rigid body with four markers ($B_{1}∼B_{4}$) was immobilized on the subject’s hand, upper arm, forearm, and torso. These rigid bodies are mainly used to solve the motion data of each joint of the upper limb. Firstly, static calibration processes were performed to solve the position of each joint center ($O_{S}$, $O_{E}$, $O_{W}$, respectively) and the transformational relationship between each rigid body’s location and the joint center. This portion of the work has been thoroughly examined in our earlier research [22,23].

(1) TJiBi=(TBiG)−1⋅TJiG, i=1,2,3,4

where TJiG and TBiG denote the joint center’s position and the rigid body’s position in global coordinates, respectively, and TJiBi denotes the transformation matrix between the rigid body and the joint center’s coordinates.

The position of the shoulder, elbow, and wrist joint centers during arm movement can be calculated using the above model. Furthermore, the kinematic characteristics of the four arm joints can be obtained:

(2) $$\theta_{4}=\pi /2-\mathrm{acos}(\frac{L_{1}^{2}+L_{2}^{2}-L_{4}^{2}}{2\cdot L_{1}\cdot L_{2}})$$

(3) $$\theta_{1}=\mathrm{atan}2(E_{x},E_{y})$$

(4) $$\theta_{2}=\mathrm{atan}2(-E_{z},\pm \sqrt{{\left(E_{x}\right)}^{2}+{\left(E_{y}\right)}^{2}})$$

(5) $$\left\{\begin{array}{c} \sin\theta_{3}=(W_{z}+s_{2}L_{1}-s_{2}s_{4}L_{2})/c_{2}c_{4}L_{2}\\ \cos\theta_{3}=(W_{x}+W_{y}-s_{1}c_{2}L_{1}+s_{1}L_{2}(c_{2}s_{4}-s_{2}s_{3}c_{4}))/c_{1}c_{4}L_{2} \end{array}\right.$$

(6) $$\theta_{3}=\mathrm{atan}2(\sin\theta_{3},\cos\theta_{3})$$

where $E(E_{x},E_{y},E_{z})$ and $W(W_{x},W_{y},W_{z})$ represent the position of the elbow and wrist joint under the coordinate system of the shoulder joint. The length of each segment of the arm is calculated using the anatomical ratio of the human upper limb, as shown in Table 1, where M is the subject’s weight.

### 2.2. Gravity Compensation Strategies

The dynamic equation of the arm can be established using the 4DOF kinematic model given in Section A:

(7) $$M_{h}(q_{h}){\ddot{q}}_{h}+C_{h}(q_{h},{\dot{q}}_{h}){\dot{q}}_{h}+G_{h}(q_{h})=\tau_{h}$$

where $\tau_{h}$ denotes the upper limb’s joint torque, $M_{h}(q_{h})$ denotes the inertial term, $C_{h}(q_{h},\;{\dot{q}}_{h})$ denotes the centrifugal and Coriolis terms, $G_{h}(q_{h})$ denotes the gravity term, and $q_{h}$ denotes the joint angle. It is worth noting that the length and weight of the forearm and hand are simplified here as a rigid linkage due to the wrist having a limited range of motion.

**Figure 2:**
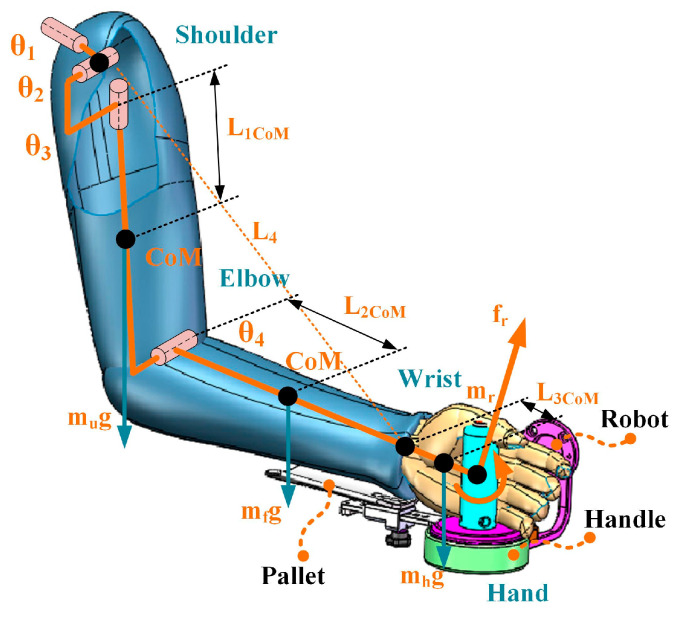
Schematic diagram of the proposed arm weight compensation strategy.

Arm weight compensation using a rehabilitation robot, such as an end-effector robot, requires applying extra force and torque at the end of the arm, as shown in Figure 2, i.e., an external wrench is added to the upper limb dynamics model to compensate for arm weight:

(8) $$M_{h}(q_{h}){\ddot{q}}_{h}+C_{h}(q_{h},{\dot{q}}_{h}){\dot{q}}_{h}+G_{h}(q_{h})=\tau_{h}+\omega_{r}(q_{h},f_{r},m_{r})$$

where $\omega_{r}(q_{h},f_{r},m_{r})$ is the external wrench applied by the robot projected in the upper limb joint space. Since the gravity torque of the subject’s arm depends on the position of the upper arm and forearm, the compensation of the gravitational term applied by the robot varies for different arm positions. The key to the proposed dynamic model-based gravity compensation strategy is to calculate the wrench that the robot applies to compensate for the gravity term $G_{h}(q_{h})$ in real time.

The quasi-static equation between the robot’s wrench applied to the end of the arm and the torque of the upper limb joint can be described as:

(9) $$\omega_{r}(q_{h},f_{r},m_{r})=J_{h}^{T}(q_{h})\left[\begin{array}{c} f_{r}\\ m_{r} \end{array}\right]$$

where $J_{h}(q_{h})$ is the Jacobian of the upper limb, ${\left[f_{r},m_{r}\right]}^{T}\in {\mathbb{R}}^{m\times 1}$ and $\omega_{r}(q_{h},f_{r},m_{r})\in {\mathbb{R}}^{p\times 1}$, where m is the robot’s DOF, and p is the arm’s DOF. The Jacobian of the arm may be constructed following the method described in our prior work [25], and the transpose of the Jacobian is guaranteed to be full-rank only when m≥p.

According to Equation (8), the external wrench applied by the robot needs to support and counteract the gravity term of the arm dynamics model:

(10) $$\omega_{r}(q_{h},f_{r},m_{r})=G_{h}(q_{h})$$

As a result, the following relationship exists between the arm gravity term and the external force and moment:

(11) $$\left[\begin{array}{c} f_{r}\\ m_{r} \end{array}\right]=J_{h}^{T}{\left(q_{h}\right)}^{\#}G_{h}(q_{h})$$

where $J_{h}^{T}{\left(q_{h}\right)}^{\#}$ is the transposed inverse matrix of the Jacobian for the human upper limb. In the established arm dynamics model (Equation (7)), the gravity term is constructed as follows:

(12) $$G_{h}(q_{h})={\left[g_{1},\cdots ,g_{n}\right]}^{T}$$

(13) gi=−∑j=1nmjg¯T∂Tj0∂qir¯j

where $m_{j}$ is the joint mass, $\bar{g}={\left[g,0\right]}^{T}$, g is the gravity acceleration vector, ${\bar{r}}_{j}$ is the coordinate of the center of mass, and Tj0 is the transformation matrix of the joint j in the shoulder joint coordinate system. The parameters $m_{j}$ and ${\bar{r}}_{j}$ can be calculated from Table 1.

However, the use of an end-effector rehabilitation robot can compensate for the end force $f_{r}$ but does not compensate effectively for the torque $m_{r}$ for the established weight compensation model. In this case, the Jacobian of the upper limb considers only the translational components of the external wrench applied by the robot. Thus, the external wrench then becomes:

(14) $$\omega_{r}(q_{h},f_{r})=J_{h-\mathrm{Tran}}^{T}(q_{h})f_{r}$$

where $J_{h-\mathrm{Tran}}^{T}(q_{h})$ is the translation components of the Jacobian for the upper limb. In this case, the external force applied by the robot for weight compensation is projected into the joint space of the arm.

(15) $$f_{r}=J_{h-\mathrm{Tran}}^{T}{\left(q_{h}\right)}^{\#}G_{h}(q_{h})$$

The system described here is underdriven because the arm in this paper is simplified as a four-DOF model, and the end-effector rehabilitation robot can only provide forces in three directions. Therefore, not all of the gravity terms in the arm dynamics model can be fully compensated. Thus, a method based on the least squares generalized inverse is adopted to minimize the error:

(16) $$\min\left\|J_{h-\mathrm{Tran}}^{T}{\left(q_{h}\right)}^{\#}G_{h}(q_{h})-f_{r}\right\|$$

**Figure 3:**
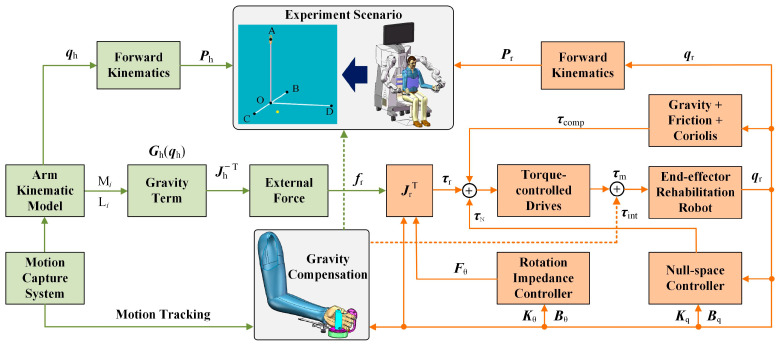
The control structure of the proposed gravity compensation strategy.

The rehabilitation robot needs to provide this external force after figuring out the end force that is employed to compensate for the arm weight. Generally, the dynamics equation of the robot with n DOF in the joint space can be expressed as:

(17) $$M_{r}(q_{r}){\ddot{q}}_{r}+C_{r}(q_{r},{\dot{q}}_{r}){\dot{q}}_{r}+G_{r}(q_{r})+F_{r}({\dot{q}}_{r})=\tau_{r}+\tau_{\mathrm{ext}}$$

where $\tau_{r}\in {\mathbb{R}}^{n\times 1}$ is the applied joint torque, $M_{r}(q_{r})\in {\mathbb{R}}^{n\times n}$ is the inertia matrix, $C_{r}(q_{r},{\dot{q}}_{r})\in {\mathbb{R}}^{n\times n}$ denotes the centrifugal and Coriolis terms; $G_{r}(q_{r})\in {\mathbb{R}}^{n\times 1}$ represents the gravity term; $F_{r}({\dot{q}}_{r})\in {\mathbb{R}}^{n\times 1}$ denotes the friction force, and $\tau_{\mathrm{ext}}\in {\mathbb{R}}^{n\times 1}$ is the external torque. Consequently, the robot’s control torque with arm weight compensation is as follows:

(18) $$\tau_{r}=M_{r}(q_{r}){\ddot{q}}_{r}+C_{r}(q_{r},{\dot{q}}_{r}){\dot{q}}_{r}+G_{r}(q_{r})+F_{r}({\dot{q}}_{r})-J_{r-\mathrm{Tran}}{\left(q_{r}\right)}^{T}f_{r}$$

where $J_{r-\mathrm{Tran}}(q_{r})$ is the translation components of the robot’s Jacobian [25]. The control flowchart of the gravity compensation strategy is shown in Figure 3.

The portion of the gravity terms in the upper limb dynamic model that are not compensated for by the weight compensation model can be expressed as:

(19) $$\begin{array}{ll} \tau_{\mathrm{uncom}} & =G_{h}(q_{h})-J_{h-\mathrm{Tran}}^{T}(q_{h})f_{r}\\ & =\left(I_{4}-J_{h-\mathrm{Tran}}^{T}(q_{h})J_{h-\mathrm{Tran}}^{T}{\left(q_{h}\right)}^{\#}\right)G_{h}(q_{h}) \end{array}$$

where $I_{4}$ is the unit matrix. According to Equation (19), the uncompensated gravity term lies in the null space of $J_{h-\mathrm{Tran}}^{T}(q_{h})$, which does not affect the linear force applied to the arm by the robot.

**Figure 4:**
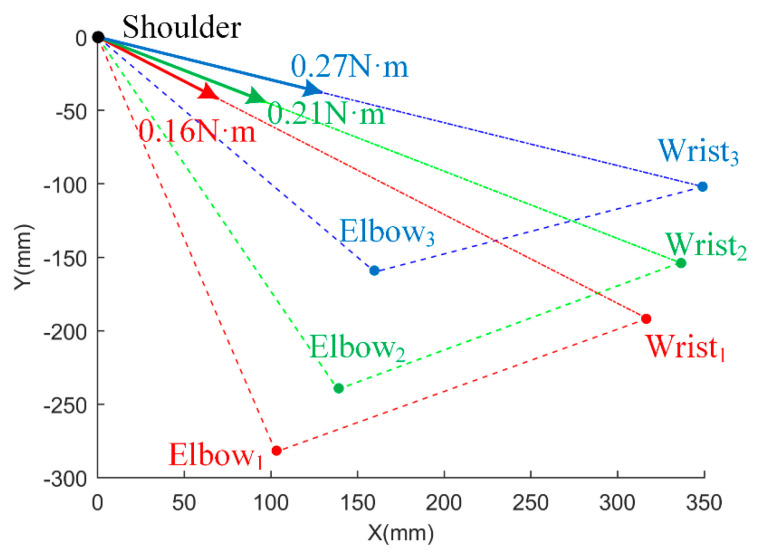
The magnitude and direction of the uncompensated torque (top view).

Three upper limb postures were chosen to describe the magnitude and direction of the uncompensated moment. The preliminary results are shown in Figure 4, where different colors (red, green, and blue) represent the three different postures, the dotted line represents the upper limb position, and the solid line represents the actual direction of the calculated uncompensated moment. As shown in Figure 4, the uncompensated portion is directed along the line connecting the shoulder and wrist joints, also known as the swivel angle axis, which is defined and used in the analysis of human–exoskeleton interactions and rehabilitation applications [15,26]. Some shoulder torque was not compensated for, whereas moments at the elbow were fully compensated in all postures, and the torque magnitude depended on the forearm and upper arm postures. Furthermore, the torque along the swivel angle axis contributes no linear velocity to the movement at the end of the arm.

**Figure 5:**
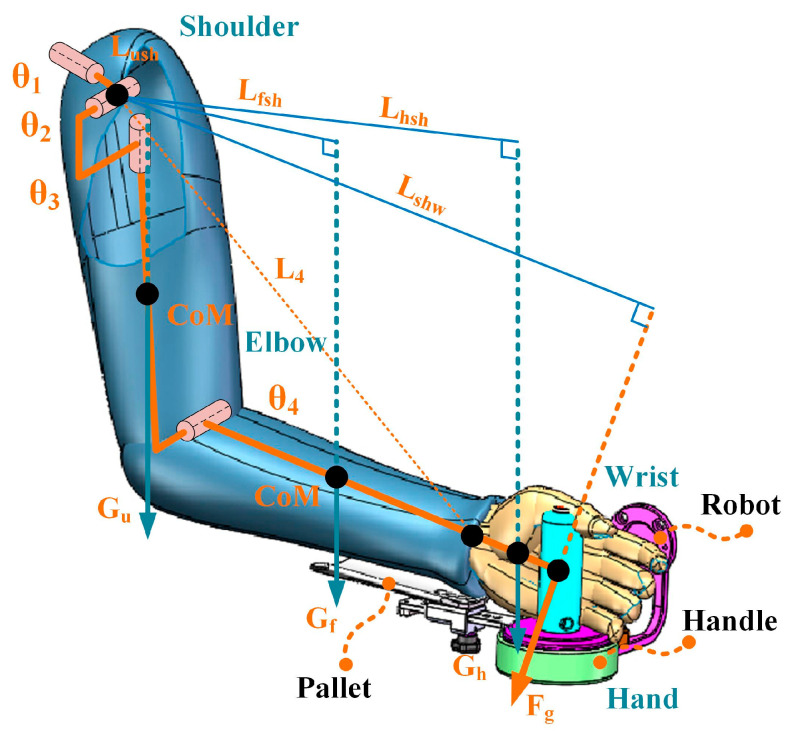
Schematic diagram of the position-varying weight compensation strategy.

There are two other weight compensation strategies besides the one proposed in this study (e.g., without compensation and position-varying weight compensation). When using a strategy without compensation, the subject must actively perform the planned movement without assistance. Another strategy is the position-varying weight compensation strategy, which is proposed in [18] and is illustrated in Figure 5, where the arm’s gravity moment is evaluated by calculating the equivalent weight at the wrist. In this method, the equivalent gravity of the weight of the upper arm, forearm, and hand at the center of the wrist is calculated, and then the combined force of these is calculated. In Figure 5, $L_{fsh}$, $L_{hsh}$, and $L_{shw}$ are the arm of force for each equivalent gravity, respectively. Finally, the weight compensation of the upper extremity is implemented by a rope-driven end-effector upper-limb rehabilitation robot.

## 3. Experiments

### 3.1. Experiment Setup

**Figure 6:**
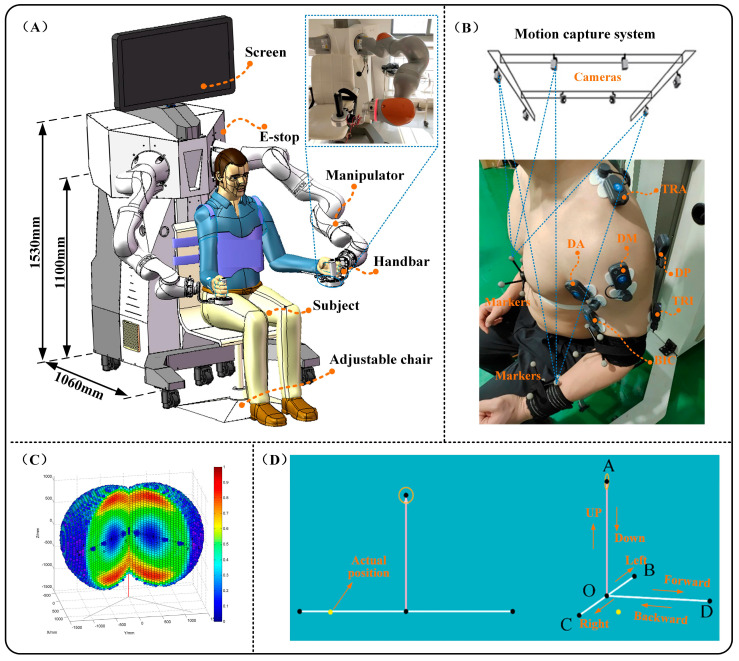
The bilateral upper limb rehabilitation robot system. (**A**) The overall structure of the system; (**B**) Motion capture system with seven cameras; (**C**) The robot’s reachability and dexterity workspace; (**D**) Schematic diagram of Experimental tasks.

The proposed weight compensation strategy is applied to an upper limb bilateral rehabilitation robot system, which is an optimized and improved version of the rehabilitation robot in the authors’ previous studies [27]. Figure 6A depicts the overall structure of the system, which mainly consists of two components: the assistance module and the ontology module. Figure 6C depicts the robot’s reachability and dexterity workspace [28]. In our experiment, the selected point-to-point task is located in the area where the robot has good dexterity. Furthermore, the FRI package allows for external torque control at frequencies up to 1000 Hz, and the friction and gravity compensation of the robot was activated throughout the experiment using the FRI package. In addition, a rotation Cartesian impedance controller was adopted to manage the robot’s pose for interaction with subjects during the experiments [29]. A null-space impedance controller is also included to manage the manipulator’s redundancy [29], which has been addressed in detail in our previous research. Furthermore, the subject’s arm and the robot were linked via a handle with a support plate, the weight of which was balanced to eliminate the effect on the experiment. The control parameters of the rehabilitation robot system during the experiment are shown in Table 2.

In addition, the system includes a motion capture system with seven cameras for reconstructing the motion of the subject’s arm, as illustrated in Figure 6B. The sampling rate of the motion capturing system was set at 100 Hz, and raw position data were filtered using a fourth-order Butterworth filter with a cutoff frequency of 6 Hz. Figure 6B shows a six-channel surface electromyographic (EMG) signal amplifier and personal computer. By attaching electrodes to the subject’s skin, the bipolar surface EMG of six superficial upper limb muscles [the biceps (BIC), the triceps (TRI), the anterior (DA), middle (DM), and posterior (DP) regions of the deltoid, and the upper trapezius (TRA)] were recorded. The EMG signals were recorded at 1000 Hz, amplified with a gain of 5000, then band-filtered with a fourth-order Butterworth filter with a 10–250 Hz bandwidth. Furthermore, the data of the motion capture system need to be unified under the world coordinate system of the robot through kinematic calibration.

### 3.2. Experimental Protocols

A total of seven able-bodied participants were recruited (mean age: 25.7 ± 1.3 years, mean weight: 64.0 ± 5.4 kg, mean height: 174.1 ± 3.8 cm). All subjects were able to lift their arms against gravity and had no musculoskeletal or neurological disorders. This study was approved by the ethics committee of the Yueyang Hospital of Integrated Traditional Chinese and Western Medicine. Written consent was collected from all subjects before participating in this study.

Before the experiment, each subject was asked to perform several motion tests to familiarize themselves with the robot system. Then, a customized rigid body with markers was worn for each subject, and surface electrodes were affixed to the protruding muscle bellies of the muscles, as illustrated in Figure 6B. The arm muscle captured in the experiment included the BIC, TRI, DA, DM, DP, and TRA, and electrode placement was performed according to the recommendations in SENIAM [30]. The collected EMG data were preprocessed using MATLAB (9.8.0.1323502, R2020a). Each subject was required to perform movement tasks with three weight compensation strategies (without weight compensation, position-varying weight compensation, and the proposed strategy) during the experiment. Each group was requested to repeat the task three times, with a 60 s pause between groups to prevent muscular tiredness, and the final result was taken as the average of the results. All subjects participated in the experiment using their left hands and were required to actively perform the task at a moderate velocity under various gravity-compensating strategies. During the experiment, a screen was placed in front of the subject to provide real-time visual feedback on the position of the hand (yellow ball), the target path (solid line), and the target point (black ball), as shown in Figure 6D. Furthermore, to reduce the impact on experimental data, reflected surfaces were avoided to the greatest extent possible at the experimental scene.

### 3.3. Data Analysis

The mean muscle activation index ($\bar{\mathrm{MAV}}$) was used in this paper to evaluate the activation levels of the subjects’ arms with different weight compensation strategies:

(20) $$\bar{\mathrm{MAV}}=\frac{\sum_{i}^{N}{\mathrm{MAV}}_{i}}{N}$$

where N is the number of samples, ${\mathrm{MAV}}_{i}$ is the envelopes of EMG signals after filtering. A smaller $\bar{\mathrm{MAV}}$ value indicates that the subject expends less effort when performing the same movement tracking [15,18].

A percentage reduction index (${\mathrm{MAV}}^{*}$) of the mean activation level of the arm muscle was proposed to compare the performance of different upper limb weight compensation strategies:

(21) $${\mathrm{MAV}}^{*}=1-\frac{{\bar{\mathrm{MAV}}}^{s}}{{\bar{\mathrm{MAV}}}^{r}}$$

where ${\bar{\mathrm{MAV}}}^{s}$ and ${\bar{\mathrm{MAV}}}^{r}$ represent the $\bar{\mathrm{MAV}}$ values after and before applying the gravity compensation strategy. The value range is [0, 1] and the greater the value, the better the performance of the weight compensation strategy.

Two-way ANOVA (analysis of variance) was chosen in this experiment to investigate the main effect of the two factors of the upper limb weight compensation strategy (without compensation, position-varying weight compensation, and the proposed weight compensation), the direction of movement, and the interaction effect of those two factors on the experimental results (mean muscle activation index). The paired *t*-test was then adopted to compare the mean muscle activation results between the three arm weight compensation strategies in different movement directions. The significance level was set at 0.05 for all statistical tests.

## 4. Results

**Figure 7:**
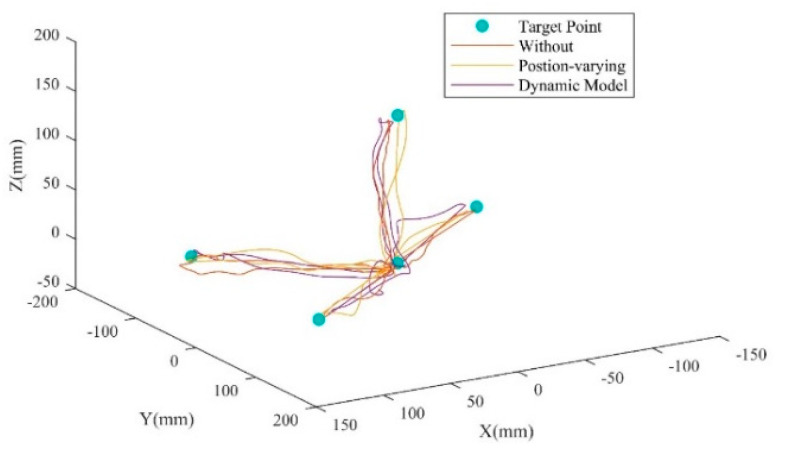
Single movement trajectories of the subjects.

Figure 7 depicts the single movement trajectories of the arm in the scenarios with different weight compensation strategies (without compensation, position-varying weight compensation, and the proposed strategy) during the experiment. ANOVA test results are shown in Table 3. The weight compensation strategy factors had a significant effect (*p* < 0.05) on the $\bar{\mathrm{MAV}}$ values of the BIC, DA, DM, DP, and TRA in this experiment, as shown in Table 3. No significant interaction between the compensation strategy and movement direction was observed.

**Figure 8:**
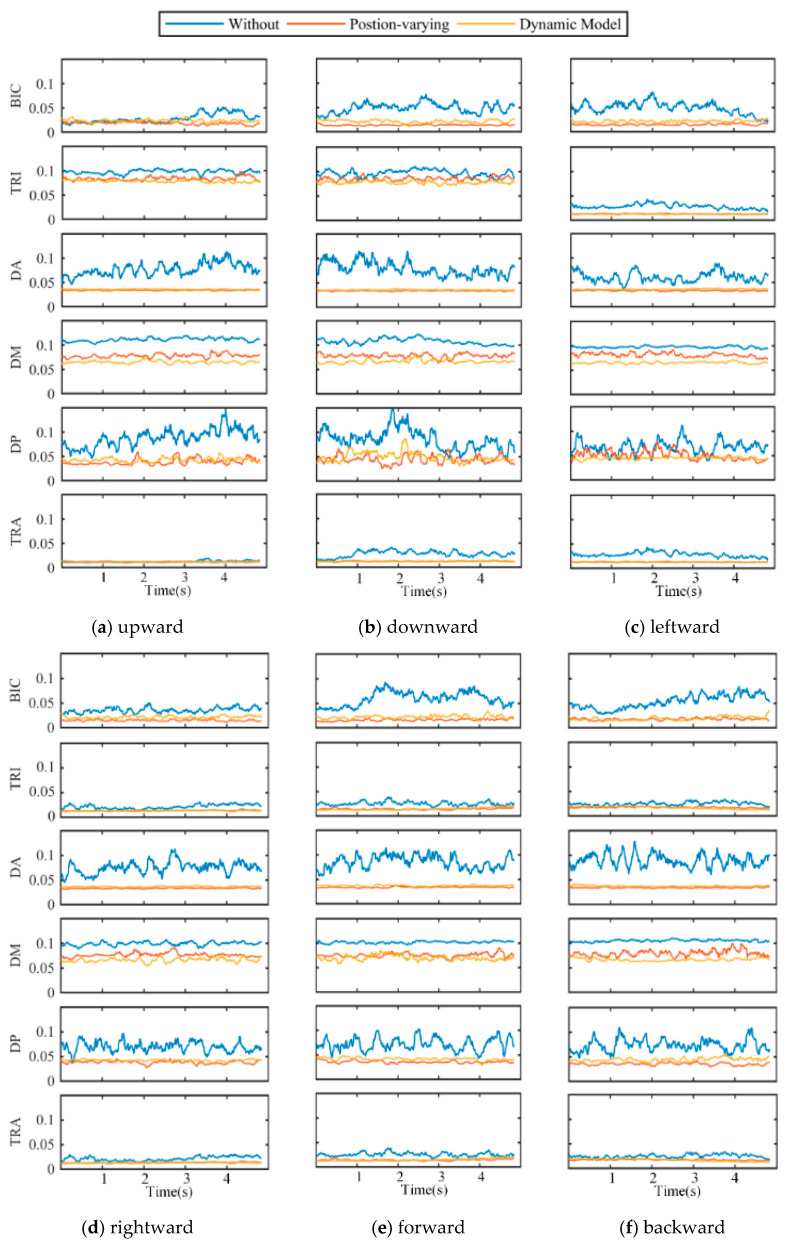
EMG envelope results of one subject for the muscles monitored during this study.

Figure 8 depicts the average EMG envelopes for six muscles under three weight compensation strategies and different movement directions for one subject. Experiment results indicated that the BIC, DA, DM, DP, and TRA muscles were less activated when subjects moved along the down, left, right, forward, and backward directions.

**Figure 9:**
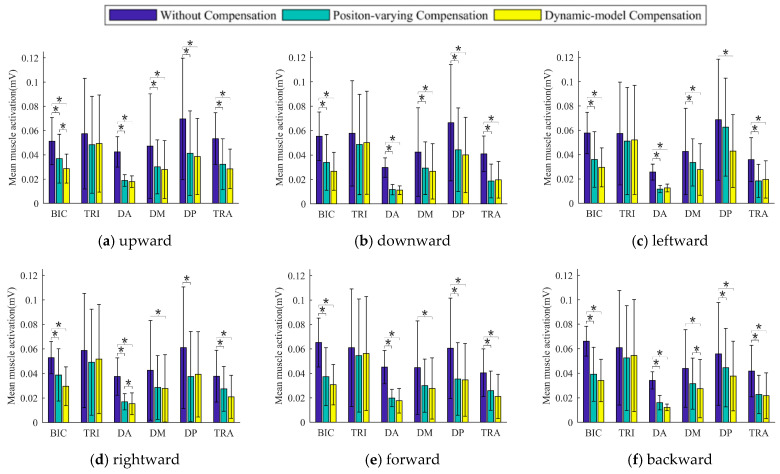
Mean $\bar{\mathrm{MAV}}$ results of the monitored muscle in different directions for all subjects. * A significant difference between two kinds of weight compensation strategies was found (*p* < 0.05).

Figure 9 depicts the $\bar{\mathrm{MAV}}$ of the muscle for all subjects using the three weight compensation strategies in six different directions. The results show that in all directions, the $\bar{\mathrm{MAV}}$ index of some muscles in the case of the proposed strategy showed significant decreases when compared to the strategy without weight compensation (e.g., the BIC, DA, DM, DP, and TRA). In contrast, the $\bar{\mathrm{MAV}}$ values of the TRI were found to be slightly lower in all directions.

**Figure 10:**
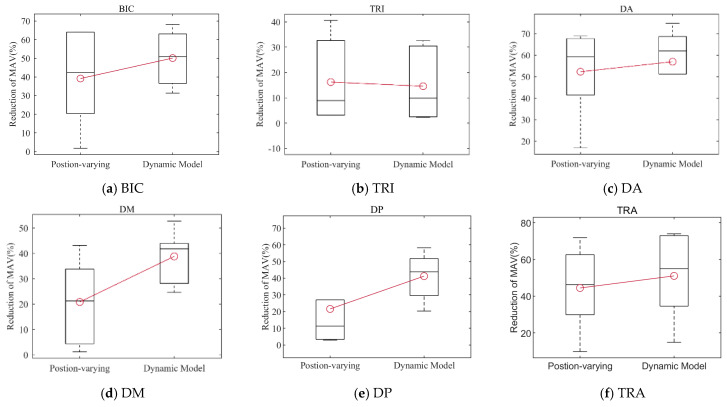
Comparative results of the mean ${\mathrm{MAV}}^{*}$ for each muscle.

Figure 10 depicts the comparison results of the mean ${\mathrm{MAV}}^{*}$ of the activation level of the arm muscle, and the box plots depict the distribution of the data in the monitored muscle for different weight compensation strategies. The mean ${\mathrm{MAV}}^{*}$ results for various weight compensation strategies are represented by the red line graphs. The results in Figure 10 show that, except for TRI, the proposed strategy was superior to the position-varying weight compensation strategy in reducing the level of muscle activation.

## 5. Discussion

The purpose of this paper is to develop a weight compensation strategy based on the arm dynamics model for an end-effector rehabilitation robot. The mean arm muscle activation levels index was adopted to evaluate and compare the performance of the proposed strategy and two other weight-compensating strategies with an end-effector upper-limb rehabilitation robot.

The $\bar{\mathrm{MAV}}$ values of the arm muscle were reduced in all directions under the gravity compensation strategy (the positon-varying compensation strategy and the proposed strategy), as shown in Figure 8 and Figure 9, compared to the movements without gravity compensation (significant reductions in the BIC, DA, DM, DP, and TRA). As the active muscle engaged in elbow flexion, the BIC is employed not only to maintain the weight of the forearm but also to help in the flexion movement of the shoulder joint. As a result, a significant decrease (*p* < 0.05) in the $\bar{\mathrm{MAV}}$ results was observed during the experiment. The deltoid muscles (DA, DM, and DP) are the primary muscles engaged in shoulder joint stabilization, as well as being active muscles involved in shoulder flexion, abduction, and internal rotation [31]. A significant decrease in $\bar{\mathrm{MAV}}$ values was also observed during the experiment (*p* < 0.05). The TRA is the primary muscle involved in lifting the upper arm and also plays a function in scapular stability and smooth shoulder joint motion [32]. In the experiment, the $\bar{\mathrm{MAV}}$ value was decreased significantly as well (*p* < 0.05). However, a non-significant decrease of $\bar{\mathrm{MAV}}$ values in the TRI was observed in this experiment, which is understandable given that the TRI is the muscle that primarily participates in elbow extension and is not the primary muscle supporting the upper limb’s weight [18]. Previous research has indicated that the BIC, DA, DP, and TRA are the primary muscles engaged in performing tasks in the horizontal plane [33]. Experimental results in the tracking task found significantly lower muscle activation levels in the BIC, DA, and DP, indicating that these are the primary muscles engaged in overcoming gravity in the upper limb [32,34]. The BIC, DA, DM, DP, and TRA were found to be the primary muscles engaged in countering gravity in the upper limb during a coronal plane task [35]. The experimental results shown in this study are compatible with the findings in previous works.

The performance of the proposed strategy and the positon-varying compensation strategy (which evaluates the arm’s gravity torque using equivalent moments) are also compared in this paper. The positon-varying compensation strategy (illustrated in Figure 5) reduces the arm to a two-link rigid body and approximates the gravity torque via the moment balance method [18]. In this paper, however, we simplify the upper limb as a 4DOF model and then evaluate the arm’s gravity torque based on the upper limb dynamics model (illustrated in Figure 2). Although both weight compensation strategies can effectively reduce arm muscle activation levels, as shown in Figure 9 and Figure 10, the mean and median ${\mathrm{MAV}}^{*}$ results of the BIC, DA, DM, DP, and TRA activation levels for the proposed strategy are higher than those of the positon-varying compensation strategy, indicating that model-based gravity compensation might better compensate for arm gravity.

### 5.1. Existing Weight Compensation Strategies

Arm weight compensation is commonly employed in robot-assisted rehabilitation. Existing robotic systems for upper limb rehabilitation, while capable of assisting the patient in resisting the arm’s gravity using mechanisms or passive devices, are either unable to implement additional strategies (e.g., Sambo [8] and ArmSpring [9]) or are unable to consider the arm’s position-coupling effects (e.g., Gentle/S [11] and Freebal [12]). However, none of these strategies enable a quantitative assessment of the subject’s arm weight. Furthermore, positive results have been achieved by applying upper limb exoskeletons to implement arm weight compensation (e.g., Harmony [13] and Armin [14,15]), which are systems that assess the arm’s gravity torque via equivalent or approximate estimations.

The assessment of gravity torque is crucial to the arm weight compensation strategy, which commonly utilizes equivalent estimation methods (e.g., the moment balance method [18,36], arm segmentation estimation methods [15], etc.). The experimental results in this paper demonstrate that the proposed weight compensation strategy can significantly lower the activation levels of the arm muscles (e.g., the BIC, DA, DM, DP, and TRA). Additionally, comparative results also indicate that the proposed strategy is superior to the positon-varying compensation strategy. This indicates that the position-varying compensation strategy proposed in [18], using the static moment balancing method, can not effectively estimate the arm’s gravitate torque. The experimental results also imply that even an end-effector-based rehabilitation robot can provide arm weight support and compensation, yielding comparable results to existing exoskeleton-based robotic systems. The experimental results also provide some references for the development of more effective and advanced arm weight compensation strategies in the future.

### 5.2. Usability of the Weight Compensation Strategies

Weight compensation resulted in a significant reduction in the average activation level of the muscles used to support the arm’s weight, implying that subjects could devote more effort to the training task. Furthermore, the activation level of the muscles corresponds to the muscle force of the arm, and a lower activation level indicates that the robot can support and compensate for the arm’s weight load, allowing the subject’s muscle force to be focused on the execution of the desired movement.

The experimental results indicate that the proposed strategy might help patients with weak muscle strength complete rehabilitation tasks. In our previous research [27,29], a variety of robot-assisted rehabilitation strategies were established to facilitate rehabilitation training. Nevertheless, the weight of the arm was regarded as an external interference. If the presented weight compensation strategy is imposed on these robot-assisted strategies, they might perform better in assisting participants in completing training tasks. The proposed strategy in this paper has two advantages. Firstly, it assists in resisting the gravity of the subject’s arm during robot-assisted movement. Secondly, there is a theoretical guarantee for evaluating arm weight torques, which provides an advantage over the static moment balance method. In addition, the weight compensation method proposed in this paper can also be applied to other assistance robot systems, such as exoskeleton-assisted robots [37].

### 5.3. Limitations

Although the work in this paper has yielded positive results, some limitations may affect the experimental outcomes and can be improved and considered in future research. Firstly, there exist some uncompensated torques when using an end-effector rehabilitation robot. However, since this portion of the torque occurs along the swivel angle axis and contributes no linear velocity to the end of the subject’s arm, it is therefore considered of secondary importance. Second, the proposed strategy has only been validated with healthy participants, and future studies should focus on more parameters and include patients or older adults to better elucidate the strategy’s performance. The proposed weight compensation method can be further optimized according to the test results. Third, additional measuring equipment or sensors, such as IMU [38], are used to calculate the exact length and mass of each subject’s arm portion to compensate for gravity, and sensitivity analysis of measurement errors to the forces is used to compensate for gravity. In addition, the multi-degree-of-freedom optimization control for these model parameters is also important and worthy of consideration in future research works.

## 6. Conclusions

In this paper, an arm weight compensation strategy was developed for an end-effector rehabilitation robot that reduces the subject’s arm muscle activation level. A 4DOF arm model was reconstructed using a motion-capture system, and then a model-based method for evaluating the arm’s gravity torque was developed. Preliminary experiment results indicate that the activation level of the arm’s main gravity-resistant muscle was significantly reduced by employing the proposed strategy compared to the method without weight compensation. The results also show that the proposed strategy is superior to the commonly used positon-varying compensation strategy in reducing the muscle’s activation level. While such an arrangement still leaves some torques along the swivel angle axis that cannot be fully compensated for, the reduced arm muscle activation level used to resist gravity suggests that the proposed weight compensation strategy has potential application for robot-assisted training. Further studies are needed to investigate the effectiveness and response of the proposed strategy in patients or older adults to validate and improve its performance.

## Figures and Tables

**Table 1:** Ratio of upper-limb length parameters [24].

	Weight	Length	Center of Mass
Upper arm	0.028 M	$L_{1}$	0.436 $L_{1}$
Forearm	0.016 M	$L_{2}$	0.430 $L_{2}$
Palms	0.006 M	$L_{3}$	0.506 $L_{3}$

**Table 2:** Control parameters of the experiments.

Variable	Values	Units
$K_{\theta }$	diag([45,45,45])	N-m/rad
$B_{\theta }$	diag([3.0,3.4,1.6])	N-m-s/rad
$K_{q}$	diag([10,10,10,10,5,5,1])	N-m/rad
$B_{q}$	diag([2.5,3.6,2.1,2.1,0.3,0.2,0.1])	N-m-s/rad
$\theta_{d}$	[−0.68,0.25,2.09]	rad
$q_{d,0}$	[−40.0,−14.8,−26.0,85.2,27.1,−34.2,−16.3]	deg

where $K_{\theta }$ and $B_{\theta }$ are the impedance parameters of the rotational impedance controller, $\theta_{d}$ is the cartesian pose (expressed with Euler angles) of the robot’s end, $K_{q}$ and $B_{q}$ are the impedance parameters of the joint impedance controller, and $q_{d,0}$ is the initial pose of the robot.

**Table 3:** The results for all factors involved in ANOVA tests.

*F*-Value		Main Effects		Interaction Effect
Outcome Measures		Compensation Method (DOF = 2)	Direction (DOF = 5)	Compensation Method × Direction (DOF = 10)
Muscle Activation	BIC	13.56 (*p* = 0.001) *	1.55 (*p* > 0.050)	0.59 (*p* > 0.050)
	TRI	1.34 (*p* > 0.050)	0.86 (*p* > 0.050)	0.55 (*p* > 0.050)
	DA	22.71 (*p* = 0.000) *	3.79 (*p* > 0.050)	1.07 (*p* > 0.050)
	DM	6.78 (*p* = 0.034) *	3.44 (*p* > 0.050)	0.77 (*p* > 0.050)
	DP	2.82 (*p* = 0.007) *	2.01 (*p* > 0.050)	1.13 (*p* > 0.050)
	TRA	9.58 (*p* = 0.008) *	2.69 (*p* > 0.050)	1.14 (*p* > 0.050)

* indicates a significant statistical difference.

## Data Availability

Data will be made available on request from the authors (email: lgzhang@usst.edu.cn).
